# CBP501 induces immunogenic tumor cell death and CD8 T cell infiltration into tumors in combination with platinum, and increases the efficacy of immune checkpoint inhibitors against tumors in mice

**DOI:** 10.18632/oncotarget.20968

**Published:** 2017-09-16

**Authors:** Keiichi Sakakibara, Takuji Sato, Donald W. Kufe, Daniel D. VonHoff, Takumi Kawabe

**Affiliations:** ^1^ CanBas Co., Ltd., Numazu, Shizuoka, Japan; ^2^ Dana-Farber Cancer Institute, Harvard Medical School, Boston, Massachusetts, USA; ^3^ Translational Genomics Research Institute (TGen), Phoenix, Arizona, USA

**Keywords:** platinum agent, immunogenic cell death, immune checkpoint inhibitor, Anti-PD-1, CD8+ T cell

## Abstract

CBP501, a calmodulin-binding peptide, is an anti-cancer drug candidate and functions as an enhancer of platinum uptake into cancer cells. Here we show that CBP501 promotes immunogenic cell death (ICD) in combination with platinum agents. CBP501 enhanced a clinically relevant low dose of cisplatin (CDDP) *in vitro* as evidenced by upregulation of ICD markers, including cell surface calreticulin exposure and release of high-mobility group protein box-1. Synergistic induction of ICD by CDDP plus CBP501 as compared to CDDP alone was confirmed in the well-established vaccination assay. Furthermore, cotreatment of CDDP plus CBP501 significantly reduced the tumor growth and upregulated the percentage of tumor infiltrating CD8^+^ T cell *in vivo*. Importantly, the antitumor effect of CDDP plus CBP501 was significantly reduced by anti-CD8 antibody treatment. Based on this novel effect of CBP501, we analyzed the combination treatment with immune checkpoint inhibitors *in vivo*. Mice treated with CBP501 in combination with CDDP and anti-PD-1 or anti-PD-L1 showed an additive antitumor effect. These results support the conclusion that CBP501 enhances CDDP-induced ICD *in vitro* and *in vivo*. The findings also support the further clinical development of the CBP501 for enhancing the antitumor activity of immune checkpoint inhibitors in combination with CDDP.

## INTRODUCTION

A calmodulin-binding peptide, CBP501, was previously described as a unique G2 checkpoint-directed agent [1] and as an enhancer of platinum uptake into cancer cells [2]. Therefore, CBP501 has been used in combination with platinum-based therapy in two Phase II clinical trials for patients with malignant pleural mesothelioma [3] and non-small cell lung carcinoma. Platinum agents are used to treat almost 50% of cancer patients [4] and, specifically, cisplatin (CDDP) is effective for the treatment of diverse solid tumors, including bladder, head and neck, lung, ovarian, and testicular cancers [5]. CDDP induces DNA crosslinking damage and thereby cancer cell death in the absence of DNA damage repair [6-7]. Cytotoxic drugs, including platinum agents, are thought to be immunosuppressive; however, recent studies show that some cytotoxic agents enhance antitumor effects by modulation of the immune system [8]. One of these effects is the induction of immunogenic cell death (ICD).

ICD is a form of apoptotic cell death associated with release of endogenous danger molecules that activate three important hallmark events [9-13]: namely, (i) cell surface exposure of the endoplasmic reticulum (ER) chaperone molecule calreticulin (CRT) that functions as an “eat-me” signal and promotes uptake of antigens associated with the dead cells by antigen-presenting cells (APCs) including dendritic cells [11-12]; (ii) release of ATP from dying cells that functions as a chemoattractant to recruit APCs and their activation; and (iii) release of high-mobility group protein box-1 (HMGB1) from dead cells that induces the maturation of APCs [13]. In particular, ER stress mediated phosphorylation of eIF2α (p-eIF2α) is required for cell surface exposure of CRT [14]; therefore, p-eIF2α is thought to be used as a biomarker for ICD induction [15]. These danger signals, in combination with cancer antigens, induce maturation of dendritic cells and can lead to an adaptive immune response against tumor cells, thereby mediating anticancer immunity [10]. Several studies show that cytotoxic drugs including methotrexate (MTX), doxorubicin and oxaliplatin (L-OHP) can induce ICD [11-14, 16]. CDDP is thought to be a non-ICD inducer due to poor induction of ER stress response [17-18]; however, several reports show that low doses of CDDP induce ER stress response [19-22].

Here we show that a clinically relevant low dose of CDDP induces the upregulation of ICD markers that is enhanced by the coadministration of CBP501 *in vitro*. The results from the well-established vaccination assay shows that CBP501 plus CDDP, but not CDDP alone, induces ICD *in vivo*. Importantly, CDDP plus CBP501 induces significant antitumor effects that involve upregulation of the percentage of CD8^+^ T cells and downregulation of the percentage of M2-type macrophages in tumor tissues. The antitumor effect induced by CDDP plus CBP501 is significantly reduced by anti-CD8 antibody treatment. Furthermore, CBP501 plus CDDP, but not CDDP, significantly enhanced the antitumor effects anti-PD-1 and anti-PD-L1.

## RESULTS

**Figure 1:**
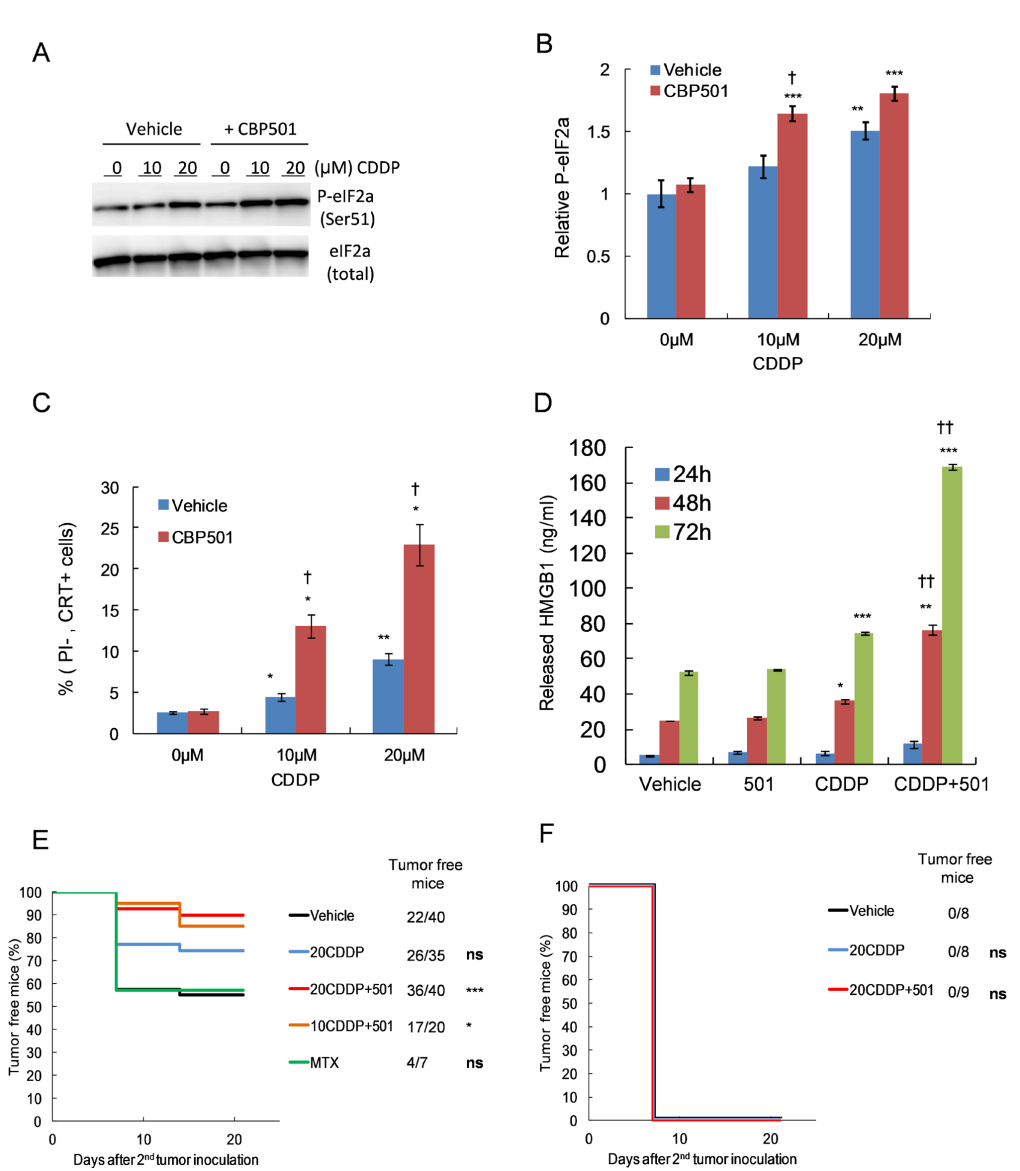
CBP501 enhances CDDP-induced immunogenic cell death both *in vitro* and *in vivo* CT26WT cells were treated with 10-20 µM CDDP in combination with 0.5 µM CBP501 for 45 min, followed by a PBS wash and addition of fresh medium. A day later, collected cells were analyzed by immunoblotting using specific antibody against phospho-eIF2-alpha (*n* = 8) **A.**-**B.** Two days later, collected cells were analyzed by FACS with specific calreticulin antibody and propidium iodide **C.** The culture medium containing released HMGB1 was collected at 24, 48 and 72 h after treatment (*n* = 3). Quantification of the secreted HMGB1 was conducted by ELISA **D.**. Treated dying CT26WT cells and live untreated cells were subcutaneously inoculated to immuneocompetent **E.** or immunodeficient **F.** mice as described in materials and methods. Tumor engraftment/ progression of the right frank were monitored once per week. Data were compared by log-rank test. Error bar indicates SEM. ^*^P < 0.05, ^**^P < 0.01, ^***^P < 0.001 compared with vehicle-treated cells, ^†^*P* < 0.05, ^††^*P* < 0.001 compared with each platinum agent-treated cells

CBP501 could enhance the antitumor activity of platinum agents including CDDP, carboplatin (CBDCA) and L-OHP and was most efficient in enhancing the activity of CDDP against CT26WT among three platinum compounds (Supplementary Figure 1). Therefore, we focused on the effect of CDDP plus CBP501. The Cmax concentrations for CDDP in blood or plasma of patients are 19-22 µM, [26-27]. We therefore used 20 µM CDDP for our *in vitro* experiments. Firstly, we measured several known ICD markers such as upregulation of p-eIF2α, cell surface expression of CRT and release of HMGB1 to determine if clinically relevant low doses of CDDP or CDDP plus CBP501 induces ICD to CT26WT cells *in vitro*. Treatment with CBP501 alone did not affect the levels of these ICD markers (Figure 1A-1D). CDDP treatment alone induced dose-dependent upregulation of p-eIF2α (Figures 1A-1B) and cell surface CRT (Figure 1C) that was further enhanced by cotreatment with CBP501. Released HMGB1, an alarm or danger signal, was gradually increased in the culture medium of vehicle- or CBP501-treated cells in time dependent manner. CDDP treatment alone slightly increased HMGB1 release; however, the combination treatment of CDDP plus CBP501 dramatically enhanced it (Figure 1D). Next, we performed the vaccination assay to evaluate the ICD induction *in vivo*. CT26WT cells treated *in vitro* with vehicle, MTX, CDDP or CDDP plus CBP501 were inoculated in the flank of immuno-competent or -deficient BALB/c mice. Induction of cell death by treatment with compounds could not be detected at this time (Supplementary Figure 2A). A week later, the mice were re-challenged with untreated CT26WT cells at 1.6 × 10^6^ cells per mice in the opposite frank. We increased three times the number of live cells (cells for secondary inoculation) from 5 × 10^5^ to 1.6 × 10^6^, because by mice inoculated with vehicle treated cells rejected the secondary inoculated tumor cells when 5 ×10^5^ live cells were used (Supplementary Figure 2B). MTX was used as a potent inducer of ICD for the vaccination assay; however, MTX-treated cells did not enhance the tumor rejection compared to vehicle-treated cells (Figure 1E), suggesting that the sensitivity of this assay was not high enough to show the effect of MTX-induced ICD on CT26WT cells to the tumor rejection. Even though the sensitivity of the vaccination assay here was not high, inoculation of the cells treated with 10 µM CDDP plus CBP501 (*P* = 0.02) and 20 µM CDDP plus CBP501 (*P* = 0.0005) induced efficient rejection of the secondary challenge of CT26WT cells much more effectively than that observed by the inoculation of cells treated with CDDP alone (*P* = 0.08), which in addition induced better tumor rejection than that of the vehicle-treated cells in immunocompetent mice (Figure 1E). The rejection did not happen for any of the conditions in immunodeficient mice (Figure 1F). These results show that a clinically relevant low dose of CDDP plus CBP501 induced ICD better than CDDP alone *in vitro* and *in vivo* and promoted tumor rejection *via* host immune system.

**Figure 2:**
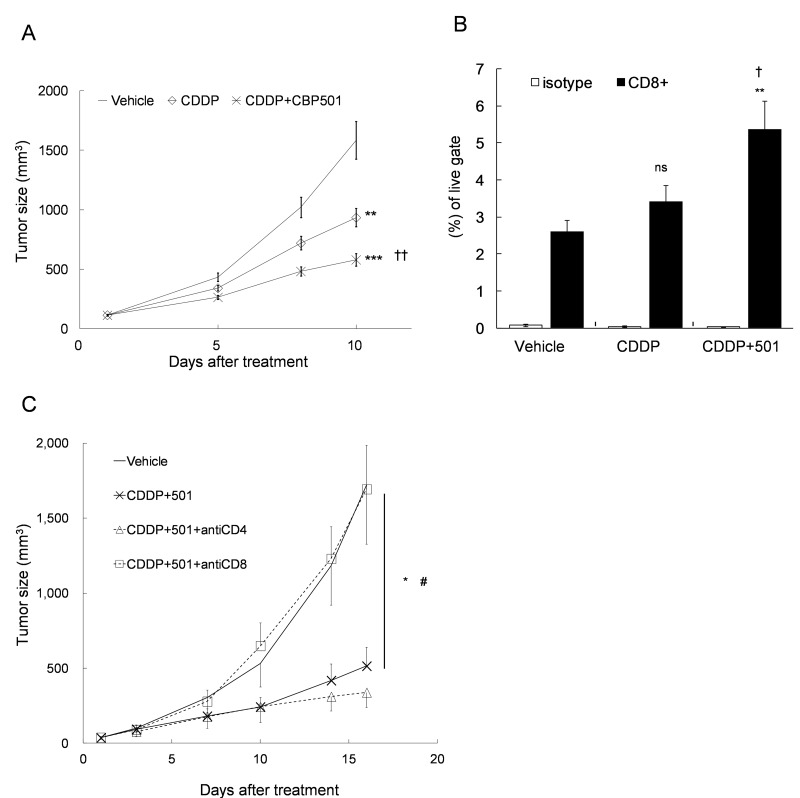
Upregulation of the percentage of CD8+ T cell in the tumor tissue by CDDP plus CBP501 treatment CT26WT cells (5 × 10^5^) were subcutaneously inoculated into BALB/c mice. Ten to eleven days later, the mice (*n* = 14) were treated intravenously with vehicle or 4 mg/kg of CDDP plus/minus 6 mg/kg of CBP501 (on days 1 and 8). The tumor growth curves **A.** and the percentage of CD8^+^ T cell in the tumor tissues on day 10 **B.** are showed. **C.** Eight days after tumor inoculation, CT26WT-tumor bearing mice (*n* = 4) were treated intraperitoneally with saline or 250 μg of anti-CD4 or anti-CD8 antibody (on days 1, 4, 7, 11 and 14) and intravenously with 4 mg/kg of CDDP plus 6 mg/kg of CBP501 (on days 3 and 10). Error bar indicates SEM. ^*^P < 0.05, ^**^P < 0.01, ^***^P < 0.0001 compared with vehicle-treated mice. ^†^*P* < 0.05, ^††^*P* < 0.001 compared with CDDP-treated mice. ^#^*P* < 0.05 compared with CDDP plus CBP501-treated mice.

Next, we evaluated the antitumor effect and the subsequent changes of tumor infiltrating lymphocytes after the treatment of CDDP plus/minus CBP501 in CT26WT implanted BALB/c mice. Intravenous administrations of CDDP alone or CDDP plus CBP501 were well tolerated, and the mean body weight losses were ∼5% or ∼10%, respectively (data not shown). CDDP treatment reduced the tumor growth that was significantly (*P* = 0.0008) enhanced by coadministration of CBP501 (Figure 2A). Analysis of tumor infiltrating cells by flow cytometry showed that treatment with CDDP plus CBP501 (*P* = 0.004), but not CDDP alone (*P* = 0.1), increased the percentage of CD8^+^ T cells (Figure 2B). When T cell subsets were depleted by function blocking anti-CD4 antibody, the antitumor effect of CDDP plus CBP501 treatment slightly enhanced; however, function blocking anti-CD8 antibody treatment significantly (*P* = 0.02) reduced the antitumor effect of CDDP plus CBP501 (Figure 2C). These results show that a clinically relevant low dose of CDDP plus CBP501 induced ICD better than CDDP alone and the importance of CD8^+^ T cell activity in the antitumor effects induced by CDDP plus CBP501 treatment.

**Figure 3:**
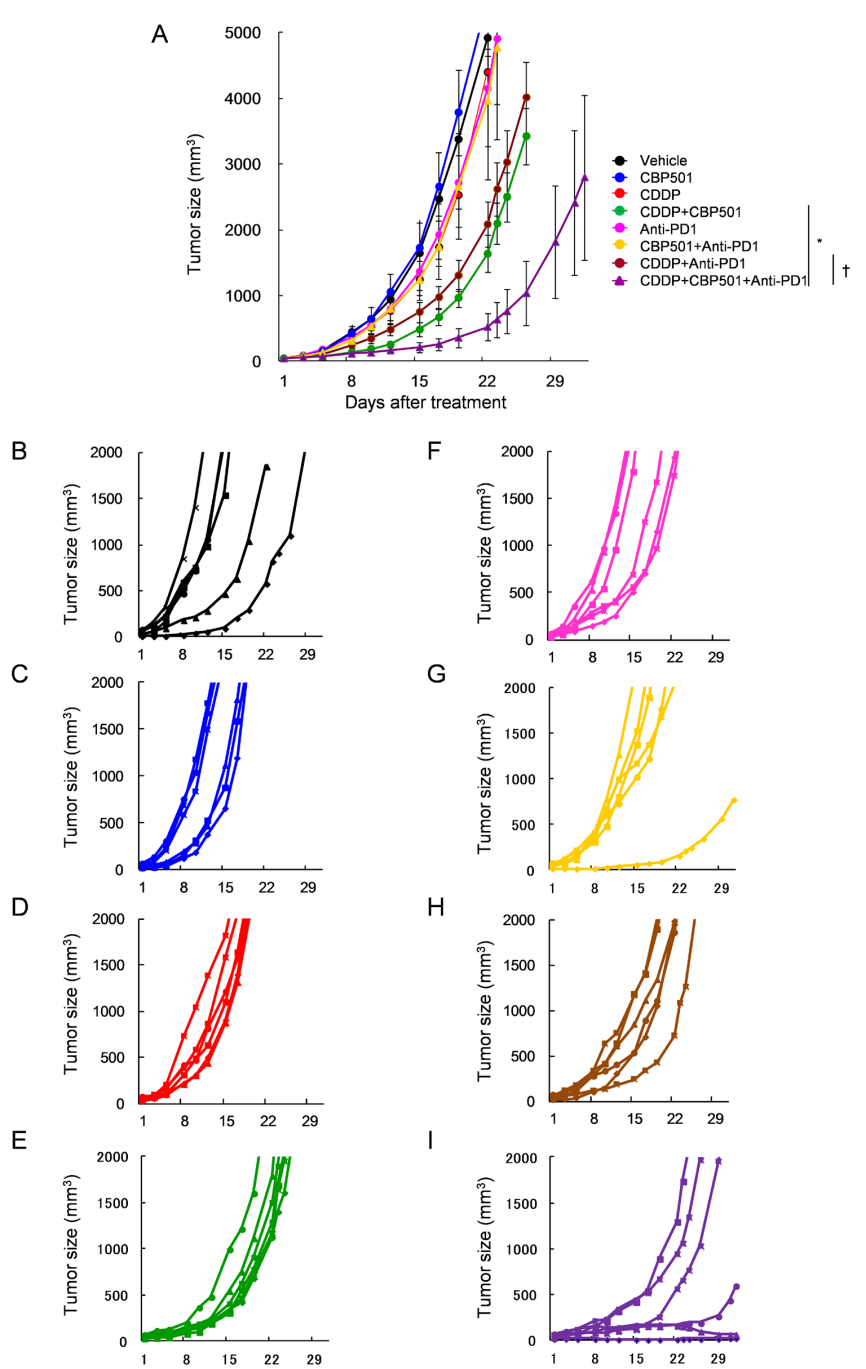
Anti-tumor effects of CDDP or CDDP plus CBP501 in combination with anti-PD-1 antibody Mice were subcutaneously inoculated into BALB/c mice. Seven days after inoculation, mice (*n* = 6) were treated intravenously with vehicle or 4 mg/kg of CDDP plus/minus 6 mg/kg of CBP501 (on days 1 and 8) and intraperitoneally with saline or 200 μg of anti-PD1 antibody (on days 2, 4, 9 and 11). Mean tumor volumes **A.** or each tumor volumes **B**.-**I**. were plotted *versus* the number of days after initiation of treatments. The groups were as follows: **B.** vehicle: black line, **C.** CBP501: blue line, **D.** CDDP: red line, **E.** CDDP + CBP501: green line, **F.** anti-PD-1: pink line, **G.** CBP501+anti-PD1 yellow line, **H.** CDDP+anti-PD1: brown line and **I.** CDDP+CBP501+anti-PD1: purple line. Error bar indicates SEM. ^*^P < 0.05 compared with CDDP+CBP501-treated mice on day22. ^†^*P* < 0.01 compared with CDDP+anti-PD1-treated mice on day22.

**Figure 4:**
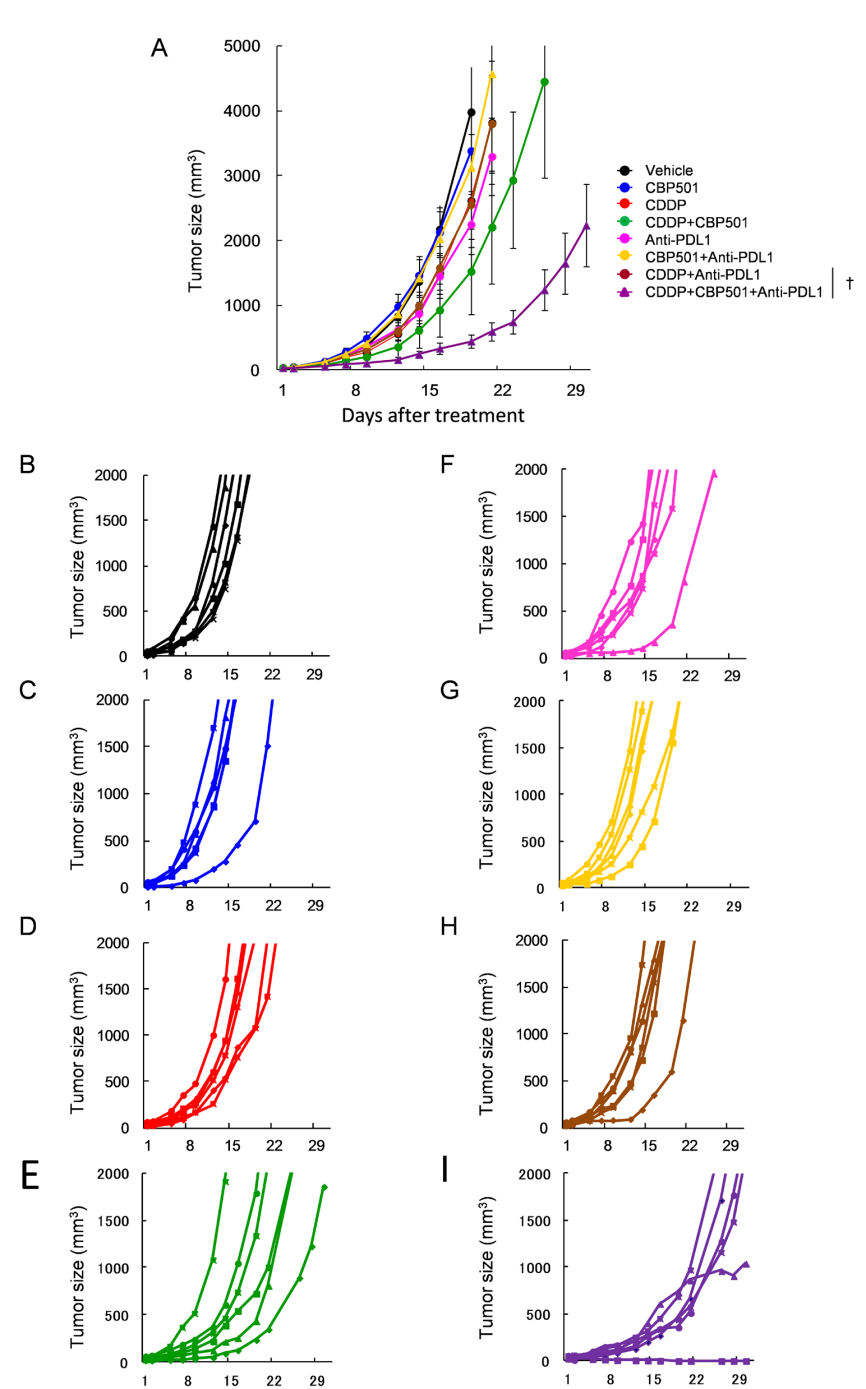
Anti-tumor effects of CDDP or CDDP plus CBP501 in combination with anti-PD-L1 antibody Mice bearing CT26WT tumor (*n* = 6) were prepared as descrived in Figure 3 and treated intravenously with vehicle or 4 mg/kg of CDDP plus/minus 6 mg/kg of CBP501 (on days 1 and 8) and intraperitoneally with saline or 200 μg of and anti-PD-L1 antibody (on days 2, 4, 9 and 11). Mean tumor volumes **A.** or each tumor volumes **B.**-**I.** were plotted *versus* the number of days after initiation of the treatment. The groups were as follows: **B.** vehicle: black line, **C.** CBP501: blue line, **D.** CDDP: red line, **E.** CDDP+CBP501: green line, **F.** anti-PD-L1: pink line, **G.** CBP501+anti-PD-L1: yellow line, **H.** CDDP+anti-PD-L1: brown line and **I.** CDDP+CBP501+anti-PD-L1: purple line. Error bar indicates SEM. ^†^*P* < 0.01 compared with CDDP+anti-PD-L1-treated mice on day21.

Based on the above mentioned findings, we evaluated the efficacy of CDDP plus/minus CBP501 when combined with immune checkpoint inhibitors, such as anti-PD-1 (Figure 3) and anti-PD-L1 (Figure 4) in CT26WT implanted BALB/c mice. Single treatments of CDDP (Figures 3D and 4D), anti-PD-1 (Figure 3F) and anti-PD-L1 (Figure 4F) but not CBP501 (Figures 3C and 4C) showed minimal antitumor effects compared to the vehicle-treated mice (Figures 3B and 4B). Combination treatments of CBP501 and CDDP (Figure 3E and 4E) effectively improved the antitumor effect of CDDP as seen in Figure 2A. Combination treatments of CBP501 and immune checkpoint inhibitors (Figures 3G and 4G) did not improve the antitumor-effect of anti-PD-1 or anti-PD-L1. The antitumor effect of anti-PD-1 but not anti-PD-L1 was enhanced by combination treatment of CDDP and immune checkpoint inhibitor (Figures 3H and 4H); however, it did not reach statistically significant level (*P* = 0.068 and *P* = 0.61, respectively) compared to immune checkpoint inhibitor alone. Triple combination treatment of CDDP plus CBP501 and anti-PD-1 (Figure 3I) or anti-PD-L1 (Figure 4I) significantly (*P* = 0.0045 and *P* = 0.0078, respectively) enhanced the antitumor effect compared to double combination treatment of CDDP and immune checkpoint inhibitors, and the tumor eradication was confirmed in some mice (Figures 3I and 4I). Similar data were obtained in combination not only with CDDP but also with CBDCA (Supplementary Figure 3).

**Figure 5:**
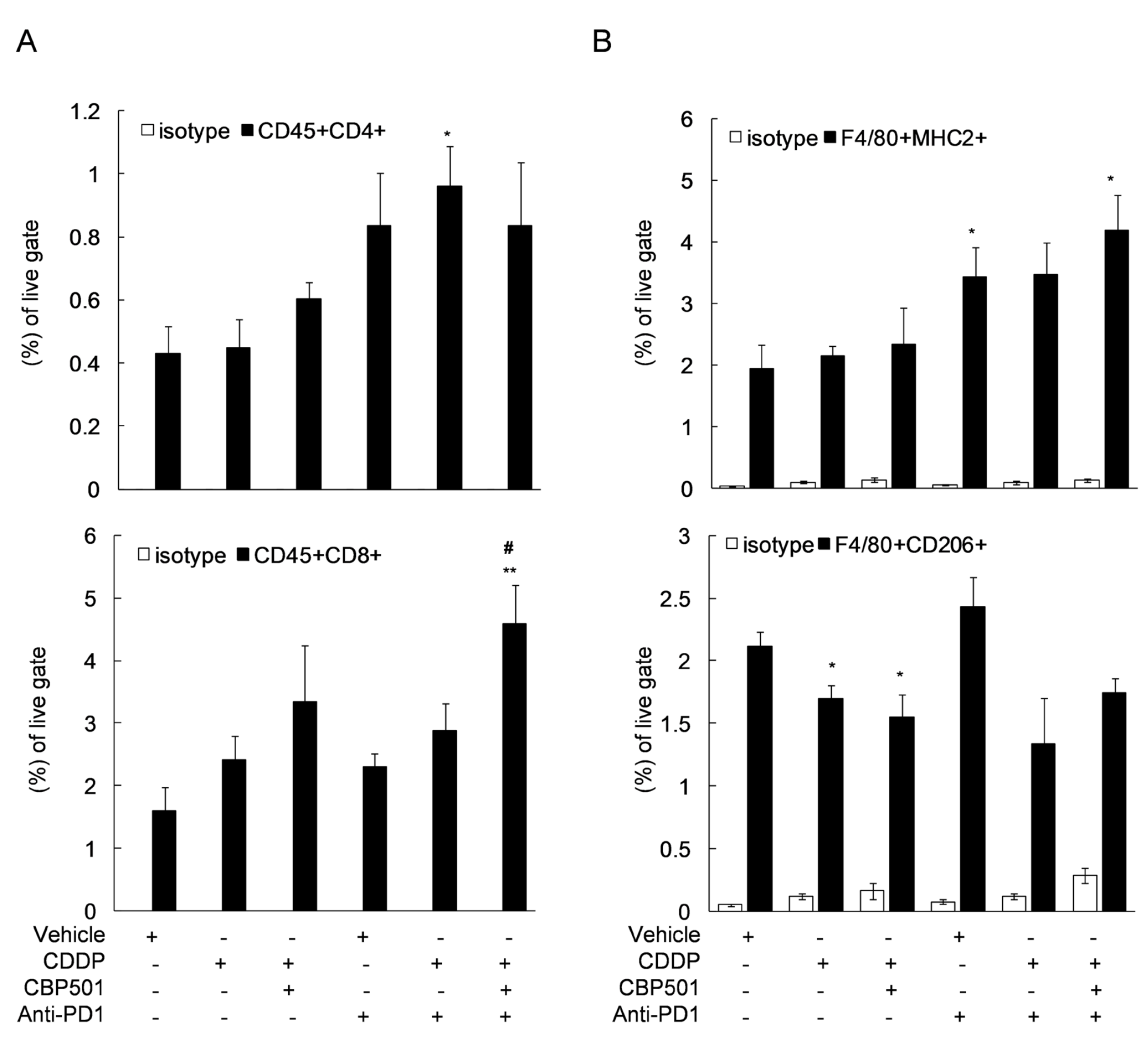
Flow cytometric analysis of tumor infiltrating-lymphocytes after triple combination treatment of CDDP plus CBP501 and anti-PD1 antibody CT26WT s.c. syngeneic mice (*n* = 4) were prepared as descrived in Figure 2A and treated as descrived in Figure 3. On day 11 after initiation of treatments, single-cell suspensions from tumor tissues were analyzed by flow cytometry using specific antibodies to monitor the percentage of T cells **A.** or TAMs **B.** in the tumor tissue. Error bar indicates SEM. ^*^P < 0.05, ^**^P < 0.01 compared with vehicle-treated mice, ^#^*P* < 0.05 compared with anti-PD1-treated mice.

Next, we evaluated the tumor infiltrating T cells (Figure 5A) and tumor associated macrophages (TAMs) (Figure 5B) in the extracted tumor tissues by flow cytometry after the treatments of CDDP, CBP501 and anti-PD-1 each alone and in their combinations. Treatment of CDDP alone and CDDP plus CBP501 tended to show increased CD8^+^ T cells as seen Figure 2B and reached statistically significant level (*P* = 0.01) with the triple combination (Figure 5A, bottom panel). The treatment of anti-PD-1 alone and triple combination of CDDP plus CBP501 with anti-PD-1 induced statistically significant increase (*P* = 0.048 and *P* = 0.023, respectively) in the ratio of M1-type TAM determined by F4/80^+^ MHCII^+^ (Figure 5B, top panel). The percentage of M2-type TAM determined by F4/80^+^ CD206^+^ was significantly downregulated by treatment of CDDP or CDDP plus CBP501 (*P* = 0.039 and *P* = 0.046, respectively), which exhibited a similar tendency even with the addition of anti-PD-1 treatment (Figure 5B, bottom panel). These results suggest that CDDP plus CBP501 induces ICD which led to the recruitment of CD8^+^ T cells into the tumor microenvironment. That effect enhanced the antitumor effects of anti-PD-1. The modulation of the ratio of TAMs also might have contributed to the antitumor activity of these combinations.

## DISCUSSION

In this report, we showed that a clinically relevant low dose of CDDP could slightly upregulate ICD markers such as p-eIF2α (Figure 1A-1B), the cell surface CRT (Figure 1C) and the HMGB1 release (Figure 1D). These responses are dramatically augmented by the cotreatment with CBP501 *in vitro*. The results of the vaccination assays confirmed that CDDP or CDDP plus CBP501 increase the immunogenicity of the treated cells (Figures 1E-1F). Tesniere *et al.* [16] reported that CDDP could not induce ICD due to its poor property to induce ER stress response; however, the dose of CDDP employed by them was 150 µM, almost ten-fold higher than the clinically relevant levels, which might have induced necrosis [28]. Gonzalez *et al.* [29] mentioned that cisplatin at high doses could damage molecules involved in cellular energy supply (i.e., ATP) and also proteins directly or indirectly involved in the apoptotic process (i.e., p53, Bax, Bcl-2, and caspases), leading to necrotic cell death. In contrast, several reports showed that low doses of CDDP induce ER stress. Mandic *et al.* [19] showed that low doses (15-20 µM) of CDDP induced upregulation of Grp78/Bip, another ER stress marker. Shi *et al.* [20] also showed that low doses of CDDP (10-40 µM) induced upregulation of Grp78/Bip and the inhibition of ER stress sensitized human lung cancer cells to CDDP. Di Blasio *et al.* [21] showed that low doses of CDDP (15 µM) treatment could induce the ICD responses including induction of p-eIF2α, cell surface CRT exposure and phagocytosis of CDDP-induced dead cell. Aranda *et al.* [22] also showed that low dose of CDDP (2.5-5 µM) plus a vitamin B6 precursor pyridoxine, which sensitizes CDDP by interfering with redox metabolism [30] could induce ICD. From these studies and our results, it can be concluded that clinically relevant doses of CDDP induce ER stress response and lead to the induction of ICD.

We used MTX as a potent inducer of ICD for the vaccination assay; however, MTX-treated cells did not enhance the tumor rejection compared to vehicle-treated cells. Aaes *et al.* [31] showed that the mice injected MTX treated CT26 cells rejected tumor engraftment at a rate about 40%. This result is similar to our data. However, they used PBS that does not contain any cells as control in the vaccination assay. When we use PBS without cells as control, 80-90% of inoculated live cell could engraft in the tumor naïve balb/c mice in our experience (data not shown). From our results, we think that vehicle treated live cells should be used as control because vehicle treated cells also induced tumor rejection to a certain extent. Even though vehicle treated cells were somewhat immunogenic, CDDP plus CBP501 treatment significantly increased the immunogenicity and promoted the rate of tumor rejection.

As CBP501 increased ICD caused by CDDP, we analyzed the tumor infiltrating CD8^+^ T cells (Figures 2B and 5A, bottom panel) and TAMs (Figure 5B) of CT26WT-tumor bearing mice after single or double combination treatment of CDDP and CBP501. Administration of CDDP plus CBP501 but not CDDP alone significantly increased CD8^+^ T cells in the tumor microenvironment (Figure 2B). This response correlated with the degree of antitumor effect (Figure 2A). Importantly, depletion of CD8^+^ T cell by a function blocking antibody led to the significant reduction of antitumor effects induced by CDDP plus CBP501 (Figure 2C). Administration of other ICD inducers, such as L-OHP plus cyclophosphamide [32] or doxorubicin [33] was reported to increase CD8^+^ T cell in the tumor tissues. These studies also support the conclusion that CDDP plus CBP501 induces ICD with increased percentages of CD8^+^ T cell in the tumor tissue.

Coadministration of immune checkpoint inhibitors, anti-PD-1 or anti-PD-L1, enhanced the antitumor effect of CDDP plus CBP501 and there was tumor eradication confirmed in about 17% of mice bearing CT26WT tumor (Figures 3-4). Flow cytometric analysis of the tumor infiltrating lymphocytes showed that the addition of CDDP plus CBP501, but not CDDP, significantly upregulated the percentage of tumor infiltrating CD8^+^ T cells induced by anti-PD-1 treatment (Figure 5A, bottom panel). The administrations of CDDP and CDDP plus CBP501 induced minimal upregulation of M1-type TAM (F4/80^+^MHC2^+^) (Figure 5B, top panel) and statistically significant downregulation of M2-type TAM (F4/80^+^CD206^+^) (Figure 5B, bottom panel), which might have contributed to the antitumor effect of CDDP plus CBP501. Interestingly, the administration of anti-PD-1 induced statistically significant upregulation of M1-type TAM (F4/80^+^MHC2^+^) (Figure 5B, top panel). These intriguing findings prompted further investigation regarding the mechanistic effects of the combination treatments of CDDP plus CBP501 with anti-PD-1. Chen *et al.* [34] reported that deficiency of PD-1 protein promotes M1 rather than M2 polarization of macrophages induced by zymosan and indicated that the PD-1 upregulation by zymosan delivered a negative signal into zymosan-stimulated bone marrow-derived macrophages, leading to the reduction of markers for M1 macrophage. It is well known that the rapid growth of tumors is accompanied by a reduced microvessel density, resulting in chronic hypoxia that often leads to necrotic areas within the tumor [35]. Therefore, anti-PD-1 might upregulate M1-type TAM through the blockade of the negative feedback signal from newly expressed PD-1 induced by HMGB1 in TAMs.

In summary, a clinically relevant low dose of CDDP slightly induced ICD which was dramatically increased by the addition of CBP501 *in vitro* and *in vivo*. The addition of CBP501 to CDDP increased CD8^+^ T cells and decreased M2-type TAMs in the tumor microenvironment in a syngeneic mice model. Noteworthy, the combination treatment of CBP501 and CDDP enhanced the antitumor activities of immune checkpoint inhibitors, anti-PD-1 or anti-PD-L1. These results provide a rationale for the use of CDDP plus CBP501 in combination with immune checkpoint inhibitors in clinical trials.

## MATERIALS AND METHODS

### Cell lines and reagents

CT26WT cells (American Type Culture Collection, CRL-2638) were maintained in RPMI1640 medium supplemented with 10% fetal bovine serum (FBS) (Biowest) and 1% Penicillin-Streptomycin (Thermo Fisher Scientific K.K., cat.#15070-063). MTX (cat.# A6770) was obtained from Sigma-Aldrich Japan. CDDP was obtained from Nippon Kayaku Co., Ltd.. Diphenhydramine was obtained from Nisshin Pharmaceutical Co., Ltd.. Saline solition was obtained from Otsuka Pharmaceuticals. Trypsin/EDTA (cat.#25200-056) was obtained from Thermo Fisher Scientific K.K.. PBS (cat.#05913) was obtained from Nissui Pharmaceutical Co., Ltd.. Function blocking antibodies including anti-PD-1 (RMP1-14, cat.#BE0146), anti-PD-L1 (10F.9G2, cat.#BE0101), anti-CD4 (GK1.5, cat.#BE0003-1) and anti-CD8 (2.43, cat.# BE0061) were obtained from Bio X Cell. All other reagents used for this study were obtained from Sigma-Aldrich Japan unless otherwise noted.

### Compound treatment *in vitro*

Cancer cells at 0.8 - 1.6 × 10^5^ were plated onto cell culture plates on the day before experiment. Next day, cells were treated with 10-20 µM CDDP plus/minus 0.5 µM CBP501 for 45 min, followed by a PBS wash and the replacement of fresh medium. Twenty four- or forty eight-hours later, cells were collected and used for specific experiments including immunoblotting and flow cytometry.

### Detection of phosphor-eIF2α

Experiments including preparation of whole cell lysates, immunoblotting, visualization of membrane bound proteins and measurement of the band intensity were performed as described previously [23]. Primary antibody for phospho-eIF2α (cat.#9721) was obtained from Cell Signaling Technology Japan k.k.. The band intensity of phospho-eIF2α was expressed as a ratio relative to that of the vehicle treated cells.

### Detection of cell surface calreticulin

The cells treated with CDDP plus/minus CBP501 were collected, and fixed with PBS containing 0.25% paraformaldehyde (Wako Pure Chemical Industries, Ltd., cat.#163-20145) for 5 min. After thrice washing with cold PBS containing 2% FBS, the cells were treated on ice with anti-calreticulin antibody (Abcam K.K., cat.#ab2907) for 30 min, followed by thrice washing with cold PBS containing 2% FBS and staining with alexa-488 conjugated donkey anti-Rabbit IgG (H+L) antibody (Thermo Fisher Scientific K.K., cat.#A-11055) for 30 min. The stained cells were washed thrice with cold PBS containing 2% FBS, treated with propidium iodide for 5 min and filtrated through a 35 µm nylon mesh prior to the flow cytometric analysis using BD FACSCalibur (BD Biosciences). Data analysis was performed by BD CellQuest software (BD Biosciences).

### Detection of released HMGB1

The medium containing released HMGB1 was collected into a microtube at 24, 48 and 72 h after treatment and kept at -80 degrees until ELISA analysis. Quantification of the released HMGB1 was conducted by HMGB1 ELISA kit II (Shino-Test Corp., cat.#3026054329) according to the manufacturer’s instructions.

### Syngeneic mice model

Female BALB/c or BALB/c-nude mice were obtained from Charles River Laboratories Japan, Inc. All animal studies were conducted according to protocols approved by institutional animal care committee of CanBas Co., Ltd. Six- to eight-week old female BALB/c mice were inoculated subcutaneously in a flank with a suspension of CT26WT cells (5 × 10^5^ cells). Seven to eleven days later mice were apportioned into 6 to 8 groups (4-6 mice/group) and treatments were initiated on day 1. Mice were intravenously treated with vehicle (saline) or CDDP (4 mg/kg) with or without CBP501 (6 mg/kg) on days 1 and 8 or days 3 and 10. Diphenhydramine (10 mg/kg) was given intraperitoneally to mice at 15 min before chemotherapy. Vehicle (saline) or immune checkpoint inhibitors including anti-PD-1 or anti-PD-L1 were given intraperitoneally to mice (200 μg/mice) on days 2, 4, 9 and 11. To deplete T cell subsets from mice, function blocking antibodies were treated as described previously [24-25] with slight modification. Anti-CD4 or anti-CD8 antibody were given intraperitoneally to mice (250 μg/mice) on days 1, 4, 7, 11 and 14. The size of tumor was measured twice or thrice weekly with a pair of calipers to calculate tumor volume by using the following formula: volume (mm^3^) = [(width)^2^ (mm) × length (mm)] / 2.

Well established vaccination assays were performed as described previously [11] with slight modification. Briefly, CT26WT cells were treated *in vitro* with vehicle, 20 µM CDDP, double combinations of 10 - 20 µM CDDP plus 0.5 μM CBP501 45 min or 2 µM MTX for 4 h, followed by a PBS wash and harvesting the cells using trypsin/EDTA. The collected cells (3 × 10^6^ cells in 200 μl of PBS) were subcutaneously inoculated in the left flank of six-week old female BALB/c or BALB/c-nude mice. A week later, live CT26WT cells (1.6 × 10^6^ cells in 100 μl of PBS) were subcutaneously inoculated again in the opposite (right) flank of the mice. Tumor engraftment/progression of the secondary inoculated tumor was monitored once per week.

### Analysis of tumor infiltrating lymphocytes

Tumor tissues from CT26WT bearing mice on day 10 or 11 after first treatment were minced and treated with triple enzyme mix [type V hyaluronidase (Sigma-Aldrich Japan, cat.#H6254), type IV collagenase and DNAse I (Worthington Biochemical Corp., cat.#LS004188 and cat.#LS002139)] for 1 h to dissociate cell from the tissue. The cell suspension was filtered through a 70 µm nylon mesh and centrifuged to precipitate the cells, followed by ACK lysis buffer treatment (155 mM NH_4_Cl, 10 mM KHCO_3_ and 0.1 mM EDTA). The cell suspension was refiltered through a 40 µm nylon mesh and the single cell suspensions (1 × 10^6^ cells) were pre-treated with anti-CD16/32 antibody (biolegend, cat.#101302) and stained with specific antibody for multi-color flow cytometry analysis, including CD4 (Biolegend, cat.#100532), CD8 (BD Biosciences, cat.#553035), CD45 (Biolegend, cat.#103106), F4/80 (Biolegend, cat.#123110), MHC class II (MHC2) (Biolegend, cat.#107616) and CD206 (Biolegend, cat.#141708) or DAPI (Thermo Fisher Scientific K.K., cat.#62248). Analysis was performed using CytoFLEX flow cytometer (Beckman Coulter K.K.), and obtained data were evaluated using FlowJo software (TOMY Digital Biology CO., LTD.)

### Statistical Analysis

The statistical significance of the differences between groups was determined by unpaired two-tailed Welch’s *t*-tests. The results of vaccination assay were analyzed by the Kaplan-Meier method and compared using the log-rank test.

## SUPPLEMENTARY MATERIALS FIGURES



## References

[1] Sha SK, Sato T, Kobayashi H, Ishigaki M, Yamamoto S, Sato H, Takada A, Nakajyo S, Mochizuki Y, Friedman JM, Cheng FC, Okura T, Kimura R (2007). Cell cycle phenotype-based optimization of G2-abrogating peptides yields CBP501 with a unique mechanism of action at the G2 checkpoint. Mol Cancer Ther.

[2] Mine N, Yamamoto S, Saito N, Yamazaki S, Suda C, Ishigaki M, Kufe DW, Von Hoff DD, Kawabe T (2011). CBP501-calmodulin binding contributes to sensitizing tumor cells to cisplatin and bleomycin. Mol Cancer Ther.

[3] Krug LM, Wozniak AJ, Kindler HL, Feld R, Koczywas M, Morero JL, Rodriguez CP, Ross HJ, Bauman JE, Orlov SV, Ruckdeschel JC, Mita AC, Fein L (2014). Randomized phase II trial of pemetrexed/cisplatin with or without CBP501 in patients with advanced malignant pleural mesothelioma. Lung Cancer.

[4] Galanski M, Jakupec MA, Keppler BK (2005). Update of the preclinical situation of anticancer platinum complexes: novel design strategies and innovative analytical approaches. Curr Med Chem.

[5] Dasari S, Tchounwou PB (2014). Cisplatin in cancer therapy: molecular mechanisms of action. Eur J Pharmacol.

[6] Huang Y, Li L (2013). DNA crosslinking damage and cancer - a tale of friend and foe. Transl Cancer Res.

[7] Siddik ZH (2003). Cisplatin: mode of cytotoxic action and molecular basis of resistance. Oncogene.

[8] Hato SV, Khong A, de Vries IJ, Lesterhuis WJ (2014). Molecular pathways: the immunogenic effects of platinum-based chemotherapeutics. Clin Cancer Res.

[9] Galluzzi L, Buqué A, Kepp O, Zitvogel L, Kroemer G (2017). Immunogenic cell death in cancer and infectious disease. Nat Rev Immunol.

[10] Vandenabeele P, Vandecasteele K, Bachert C, Krysko O, Krysko DV (2016). Immunogenic Apoptotic Cell Death and Anticancer Immunity. Adv Exp Med Biol.

[11] Casares N, Pequignot MO, Tesniere A, Ghiringhelli F, Roux S, Chaput N, Schmitt E, Hamai A, Hervas-Stubbs S, Obeid M, Coutant F, Métivier D, Pichard E (2005). Caspase-dependent immunogenicity of doxorubicin-induced tumor cell death. J Exp Med.

[12] Obeid M, Tesniere A, Ghiringhelli F, Fimia GM, Apetoh L, Perfettini JL, Castedo M, Mignot G, Panaretakis T, Casares N, Métivier D, Larochette N, van Endert P (2007). Calreticulin exposure dictates the immunogenicity of cancer cell death. Nat Med.

[13] Apetoh L, Ghiringhelli F, Tesniere A, Obeid M, Ortiz C, Criollo A, Mignot G, Maiuri MC, Ullrich E, Saulnier P, Yang H, Amigorena S, Ryffel B (2007). Toll-like receptor 4-dependent contribution of the immune system to anticancer chemotherapy and radiotherapy. Nat Med.

[14] Panaretakis T, Kepp O, Brockmeier U, Tesniere A, Bjorklund AC, Chapman DC, Durchschlag M, Joza N, Pierron G, van Endert P, Yuan J, Zitvogel L, Madeo F (2009). Mechanisms of pre-apoptotic calreticulin exposure in immunogenic cell death. EMBO J.

[15] Kepp O, Semeraro M, Bravo-San Pedro JM, Bloy N, Buqué A, Huang X, Zhou H, Senovilla L, Kroemer G, Galluzzi L (2015). eIF2α phosphorylation as a biomarker of immunogenic cell death. Semin Cancer Biol.

[16] Tesniere A, Schlemmer F, Boige V, Kepp O, Martins I, Ghiringhelli F, Aymeric L, Michaud M, Apetoh L, Barault L, Mendiboure J, Pignon JP, Jooste V (2010). Immunogenic death of colon cancer cells treated with oxaliplatin. Oncogene.

[17] Martins I, Kepp O, Schlemmer F, Adjemian S, Tailler M, Shen S, Michaud M, Menger L, Gdoura A, Tajeddine N, Tesniere A, Zitvogel L, Kroemer G (2011). Restoration of the immunogenicity of cisplatin-induced cancer cell death by endoplasmic reticulum stress. Oncogene.

[18] Michaud M, Sukkurwala AQ, Di Sano F, Zitvogel L, Kepp O, Kroemer G (2014). Synthetic induction of immunogenic cell death by genetic stimulation of endoplasmic reticulum stress. Oncoimmunology.

[19] Mandic A, Hansson J, Linder S, Shoshan MC (2003). Cisplatin induces endoplasmic reticulum stress and nucleus-independent apoptotic signaling. J Biol Chem.

[20] Shi S, Tan P, Yan B, Gao R, Zhao J, Wang J, Guo J, Li N, Ma Z (2016). ER stress and autophagy are involved in the apoptosis induced by cisplatin in human lung cancer cells. Oncol Rep.

[21] Di Blasio S, Wortel IM, van Bladel DA, de Vries LE, Duiveman-de Boer T, Worah K, de Haas N, Buschow SI, de Vries IJ, Figdor CG, Hato SV (2016). Human CD1c(+) DCs are critical cellular mediators of immune responses induced by immunogenic cell death. Oncoimmunology.

[22] Aranda F, Bloy N, Pesquet J, Petit B, Chaba K, Sauvat A, Kepp O, Khadra N, Enot D, Pfirschke C, Pittet M, Zitvogel L, Kroemer G (2015). Immune-dependent antineoplastic effects of cisplatin plus pyridoxine in non-small-cell lung cancer. Oncogene.

[23] Sakakibara K, Saito N, Sato T, Suzuki A, Hasegawa Y, Friedman JM, Kufe DW, VonHoff DD, Iwami T, Kawabe T (2011). CBS9106 is a novel reversible oral CRM1 inhibitor with CRM1 degrading activity. Blood.

[24] Zhou P, L’italien L, Hodges D, Schebye XM (2007). Pivotal roles of CD4+ effector T cells in mediating agonistic anti-GITR mAb-induced-immune activation and tumor immunity in CT26 tumors. J Immunol.

[25] Yin T, Wang G, Ye T, Wang Y (2016). Sulindac, a non-steroidal anti-inflammatory drug, mediates breast cancer inhibition as an immune modulator. Sci Rep.

[26] Kroep JR, Smit EF, Giaccone G, Van der Born K, Beijnen JH, Van Groeningen CJ, Van der Vijgh WJ, Postmus PE, Pinedo HM, Peters GJ (2006). Pharmacology of the paclitaxel-cisplatin, gemcitabine-cisplatin, and paclitaxel-gemcitabine combinations in patients with advanced non-small cell lung cancer. Cancer Chemother Pharmacol.

[27] Dijkgraaf EM, Heusinkveld M, Tummers B, Vogelpoel LT, Goedemans R, Jha V, Nortier JW, Welters MJ, Kroep JR, van der Burg SH (2013). Chemotherapy alters monocyte differentiation to favor generation of cancer-supporting M2 macrophages in the tumor microenvironment. Cancer Res.

[28] Sancho-Martínez SM, Piedrafita FJ, Cannata-Andía JB, López-Novoa JM, López-Hernández FJ (2011). Necrotic concentrations of cisplatin activate the apoptotic machinery but inhibit effector caspases and interfere with the execution of apoptosis. Toxicol Sci.

[29] Gonzalez VM, Fuertes MA, Alonso C, Perez JM (2001). Is cisplatin-induced cell death always produced by apoptosis?. Mol Pharmacol.

[30] Galluzzi L, Vitale I, Senovilla L, Olaussen KA, Pinna G, Eisenberg T, Goubar A, Martins I, Michels J, Kratassiouk G, Carmona-Gutierrez D, Scoazec M, Vacchelli E (2012). Prognostic impact of vitamin B6 metabolism in lung cancer. Cell Rep.

[31] Aaes TL, Kaczmarek A, Delvaeye T, De Craene B, De Koker S, Heyndrickx L, Delrue I, Taminau J, Wiernicki B, De Groote P, Garg AD, Leybaert L, Grooten J (2016). Vaccination with Necroptotic Cancer Cells Induces Efficient Anti-tumor Immunity. Cell Rep.

[32] Pfirschke C, Engblom C, Rickelt S, Cortez-Retamozo V, Garris C, Pucci F, Yamazaki T, Poirier-Colame V, Newton A, Redouane Y, Lin YJ, Wojtkiewicz G, Iwamoto Y (2016). Immunogenic chemotherapy sensitizes tumors to checkpoint blockade therapy. Immunity.

[33] Kawano M, Tanaka K, Itonaga I, Iwasaki T, Miyazaki M, Ikeda S, Tsumura H (2016). Dendritic cells combined with doxorubicin induces immunogenic cell death and exhibits antitumor effects for osteosarcoma. Oncol Lett.

[34] Chen W, Wang J, Jia L, Liu J, Tian Y (2016). Attenuation of the programmed cell death-1 pathway increases the M1 polarization of macrophages induced by zymosan. Cell Death Dis.

[35] Kostova N, Zlateva S, Ugrinova I, Pasheva E (2010). The expression of HMGB1 protein and its receptor RAGE in human malignant tumors. Mol Cell Biochem.

