# Supplementary material for: CBP501 induces immunogenic tumor cell death and CD8 T cell infiltration into tumors in combination with platinum, and increases the efficacy of immune checkpoint inhibitors against tumors in mice
Source: Oncotarget. 2017 Sep 16;8(45):78277–88. doi: 10.18632/oncotarget.20968 (PMC5663279; doi:10.18632/oncotarget.20968)
Supplement: Supplementary file 1 [file oncotarget-08-78277-s001.pdf]

CBP501 induces immunogenic tumor cell death and CD8 T cell infiltration into tumors in combination with platinum, and increases the efficacy of immune checkpoint inhibitors against tumors in mice

SUPPLEMENTARY MATERIALS

Syngeneic mice model

Carboplatin (CBDCA) was obtained from SANDOZ K.K.. Six- to eight-week old female BALB/c mice were inoculated subcutaneously in a flank with a suspension of CT26WT cells (5 x 10^5 cells). Seven to eight days later mice were apportioned into 6 groups (6-9 mice/group) and treatments were initiated on day 1. Mice were intravenously treated with vehicle (saline) or CBDCA (50 mg/kg) with or without CBP501 (6 mg/kg) on days 1 and 8. Diphenhydramine (10 mg/kg) was given intraperitoneally to mice at 15 min before chemotherapy. Vehicle (saline) or anti-PD-L1 were given intraperitoneally to mice (400 µg/mice) on days 4, 11 and 16.

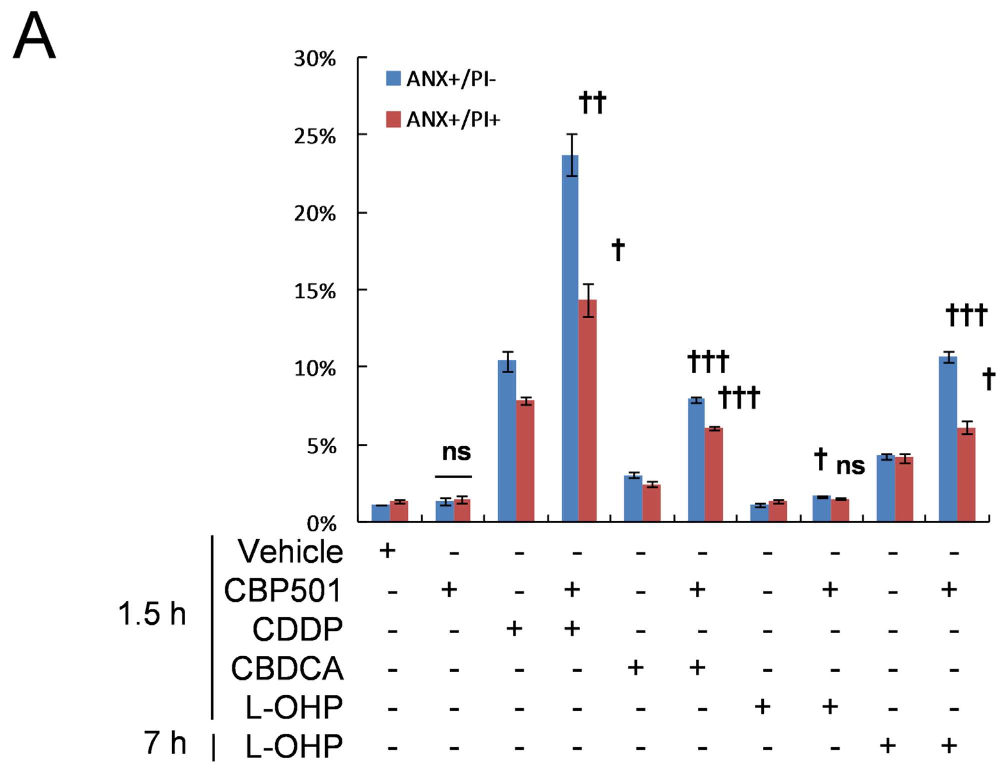

Supplementary Figure 1: CBP501 enhances cell death induced by platinum agents. A murine colorectal cell line, CT26WT cells were treated clinically relevant concentrations of platinum agents, 20 µM CDDP, 200 µM CBDCA and 15 µM L-OHP in combination with 0.5 µM CBP501 for 1.5 h or 7 h, followed by PBS wash and addition of fresh medium. Two days later, induction of cell death was analyzed by annexin V kits. Data are means±SEM. Data were compared by unpaired two-tailed Welch's t tests. $^{*}p < 0.05$, $^{**}p < 0.01$, $^{***}p < 0.001$, compared with vehicle-treated cells, and $^{†}p < 0.05$, $^{††}p < 0.01$, $^{†††}p < 0.001$, compared with corresponding platinum agent-treated cells.

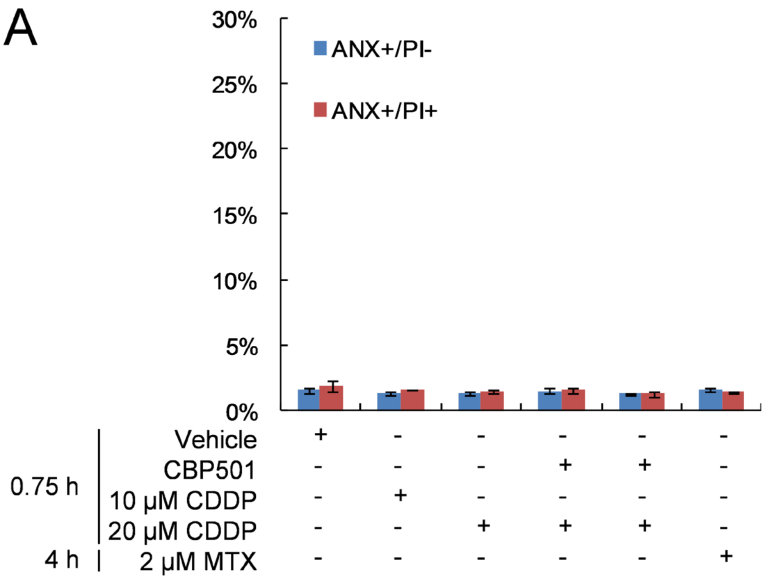

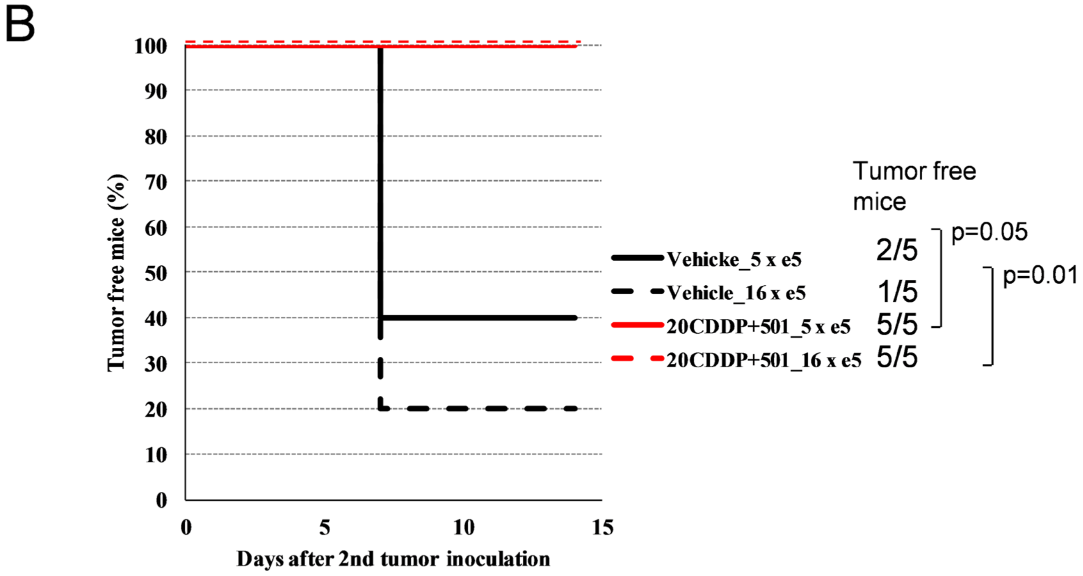

Supplementary Figure 2: Evaluation of cell number for secondary inoculation of CT26 live cells in vaccination assay (A) CT26WT cells were treated with 2 μM MTX for 4 h and vehicle or double combination of 20 μM CDDP plus 0.5 μM CBP501 for 45min, followed by collection of cells using trypsin/EDTA. The collected cells were analyzed by annexin V kits. Data are means±SEM. **(B)** The CT26WT cells treated with vehicle or double combination of 20 μM CDDP plus CBP501 for 45min (3 x 10^6 cells) were subcutaneously inoculated in left frank of BALB/c mice. A week later, live CT26 cells (5.0 x 10^5 or 1.6 x 10^6 cells) were subcutaneously inoculated in the right frank of BALB/c mice. Tumor engraftment/ progression of the right frank were monitored once per week. Data were compared by log-rank test.

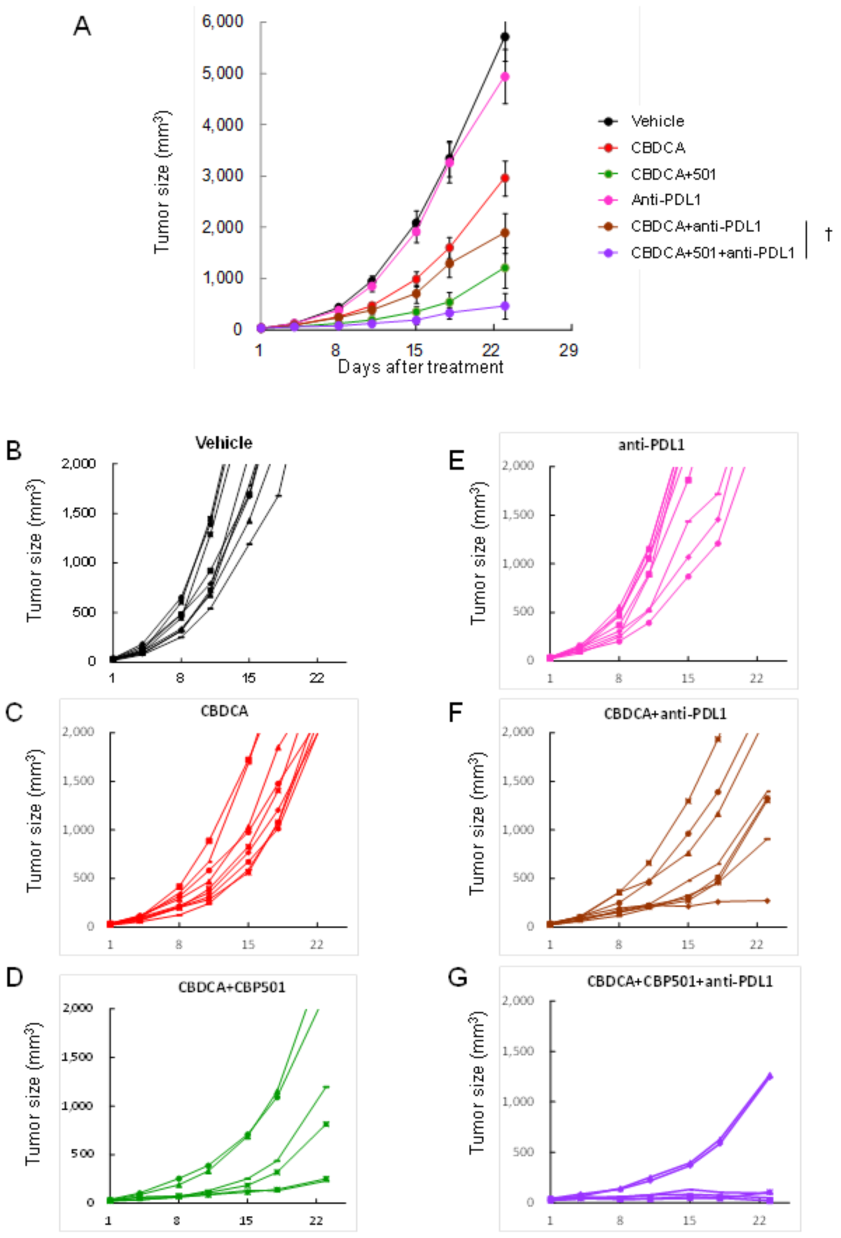

Supplementary Figure 3: Anti-tumor effects of CBDCA or CBDCA plus CBP501 in combination with anti-PD-L1 antibody. Mice bearing CT26WT tumor (n=6-9) were treated intravenously with vehicle or 50 mg/kg of CBDCA plus/minus 6 mg/kg of CBP501 (on days 1 and 8) and intraperitoneally with saline or 400 µg of anti-PD1 antibody (on days 4, 11 and 16). Mean- **(A)** or each- (**B-G**) tumor volumes were plotted versus the number of days after initiation of treatments. The groups were as follows: **(B)** vehicle: black line, **(C)** CBDCA red line, **(D)** CBDCA+CBP501: green line, **(E)** anti-PD-L1: pink line, **(F)** CBDCA+anti-PD-L1: brown line and **(G)** CBDCA+CBP501+anti-PD-L1: purple line. Error bar indicates SEM. †P<0.01, compared with CBDCA+anti-PD-L1-treated mice on day23.
